# Alendronate increases BMD at appendicular and axial skeletons in patients with established osteoporosis

**DOI:** 10.1186/1749-799X-2-9

**Published:** 2007-05-21

**Authors:** Ling Qin, Wingyee Choy, Szeki Au, Musei Fan, Pingchung Leung

**Affiliations:** 1 Department of Orthopaedics and Traumatology, The Chinese University of Hong Kong, Hong Kong SAR, China; 2 Hong Kong Jockey Club Center for Osteoporosis Care and Control, The Chinese University of Hong Kong, Hong Kong SAR, China

## Abstract

**Background:**

To identify high-risk patients and provide pharmacological treatment is one of the effective approaches in prevention of osteoporotic fractures. This study investigated the effect of 12-month Alendronate treatment on bone mineral density (BMD) and bone turnover biochemical markers in postmenopausal women with one or more non-traumatic fractures, i.e. patients with established osteoporosis.

**Methods:**

A total of 118 Hong Kong postmenopausal Chinese women aged 50 to 75 with low-energy fracture at distal radius (Colles' fracture) were recruited for BMD measurement at lumbar spine and non-dominant hip using Dual-Energy X-ray Absorptiometry (DXA). 47 women with BMD T-score below -2 SD at either side were identified as patients with established osteoporosis and then randomized into Alendronate group (n = 22) and placebo control group (n = 25) for BMD measurement at spine and hip using DXA and distal radius of the non-fracture side by peripheral quantitative computed tomography (pQCT), and bone turnover markers, including bone forming alkaline phosphatase (BALP) and bone resorbing urinary Deoxypyridinoline (DPD). All measurements were repeated at 6 and 12 months.

**Results:**

Alendronate treatment significantly increased BMD, more in weight-bearing skeletons (5.1% at spine and 2.5% at hip) than in non-weight bearing skeleton (0.9% at distal radius) after 12 months treatment. Spine T-score was significant improved in Alendronate group (p < 0.01) (from -2.2 to -1.9) but not in control placebo group. The Alendronate treatment effect was explained by significant suppression of bone turnover.

**Conclusion:**

12 months Alendronate treatment was effective to increase BMD at both axial and appendicular skeletons in postmenopausal women with established osteoporosis.

## Background

Our recent retrospective study shows that postmenopausal women with low-energy Colles' fractures are associated with osteoporosis [1]. Similar studies are also reported before that osteoporotic fracture is often seen in high risk patients such as those with established osteoporosis, i.e. osteoporotic patients with one or more low-energy fractures [2-4]. Osteoporotic fracture incurs high morbidity, mortality and healthcare expenditure [3,5-7]. The current consensus for effective prevention of osteoporotic fracture is to identify the high-risk patients and put them on effective pharmacological intervention programs.

Anti-resorptive drugs such as Bisphosphonates have been proven to be effective for treatment of osteoporosis and fracture prevention in patients with osteoporosis [7-10]. The aim of this study was to evaluate effects of 12-month Alendronate treatment in postmenopausal women with established osteoporosis. Bone mineral density (BMD) was used as the end-point and bone turnover biochemical markers were evaluated for monitoring bone metabolism in response to drug treatment effects.

## Methods

In order to confirm our treatment effects, we identified patients with established osteoporosis for treatment and used bone mineral density (BMD) at both axial and appendicular skeletons as the end-point for evaluations.

### Subject recruitment and identification of patients with established osteoporosis

One hundred eighteen postmenopausal women, aged 50–75 with one low-energy fracture at distal radius (Colles' fracture) in the past 5 years, were concurrently recruited form the hospital of the investigators as described in our recent study [1]. Each author certifies that his or her institution has approved the human protocol for this investigation and that all investigations were conducted in conformity with ethical principles of research, and that informed consent was obtained. Exclusion criteria were women under hormonal replacement therapy or drug treatment known to affect bone metabolism, with condition such as hypo- or hyperparathyroidism and hypo- or hyperthyroidism, renal or liver disease. In order to avoid the possible adverse effect of Alendronate on gastrointestinal tract, women with history of gastrointestinal tract disease or chronic stomach disease were also excluded [8,9]. All subjects had BMD measurement at lumbar spine (L2-L4) and non-dominant hip (femoral neck) by Dual-Energy X-ray Absorptiometry (DXA) (Norland XR36, Norland Corporation, Fort Atkinson, WI, USA). Patients with established osteoporosis were those found to have T-score below -2 SD at spine or hip [1,6,11]. Finally, a total of around 40%, i.e. 47 subjects were identified and recruited as patients with established osteoporosis and randomized into Alendronate treatment group (n = 22) and placebo control group (n = 25). Body height and body weight were measured and body mass index (BMI, kg/m^2^) was calculated.

### Treatments

10 mg alendronate was used for subjects in Alendronate group, together with 1200 mg calcium supplement per day, as it dose was reported as an effective dose for the same study population for Hong Kong Chinese [8,9]. Control group was given 'placebo tablets', i.e.1200 mg calcium supplement per day. The intervention lasted 12 months.

### Monitoring treatment effects

In order to investigate systemic treatment effects, clinical important axial and appendicular skeletal sides prone to osteoporotic fractures were selected for BMD measurement, including areal BMD (g/cm^2^) at spine and hip measured by DXA and volumetric BMD (g/cm^3^) of the non-fracture distal radius using a multilayer peripheral quantitative computed tomography (pQCT) (Densiscan 2000, Scanco Medical, Bassersdorf, Switzerland). For pQCT measurement, a standard program with 16 tomographs was used. Thickness of each layer is 1 mm with 1.5 mm interval between each other. Trabecular BMD (tBMD) in a core volume (central 50% area of total bone area) and integral BMD (iBMD) within the total volume of the ultradistal radius were obtained from the first ten distal tomographs. Cortical BMD (cBMD) was obtained from the pure cortical compartment of distal disphysis from the six proximal tomographs. Technical details are described previously [1,12]. Quality control scans for both DXA and pQCT were performed daily with a manufacture-supplied anthropometric phantom, which showed a precision error of 1.2% for DXA and 0.3% for pQCT reported for the same reference population [1,12].

### Bone turnover biochemical markers

Biochemical markers were used to monitor the changes of bone turnover after Alendronate treatment and compared with placebo control group. These included serum bone specific alkaline phosphatase (BALP) as a bone formation marker by collecting the blood sample at the same day time and urinary Deoxypyridinoline (DPD) as a bone resorption marker by collecting urine as the first morning void sample on the same day. Both blood and urine samples were then stored in -80 C freezer before biochemical assay. BALP was measured with a specific lectin precipitation method using autoanalyser (Abbott VP system) and DPD was measured by commercial available ELISA kit PYRILINKS-D (Metra Biosystem, USA) [8,13]. Serum BALP and urinary DPD were measured at both baseline and follow-up at 6 and 12 months.

### Dropout and facture case

Both number and reason of dropout was recorded. Fracture cases during 12-month treatment period was also recorded and confirmed radiographically.

### Statistics

Un-paired T-test was used to study the homogeneity on the randomization of two groups at baseline. ANOVA was used to detect the difference in BMD at both axial and appendicular skeletons at baseline, 6 and 12 months for each group and between two groups. The statistical significance was set at p < 0.05. SPSS 11.0 statistical program (444 North Michigan Avenue, Chicago, IL 60611, USA) was used for data analysis.

## Results

### Randomization of subjects for two study groups

Table 1 shows the homogeneity in anthropometric variables (age, YSM, body height and weight, and BMI) and DXA T-score compared between Alendronate group and placebo control group before starting intervention. 39.8% (47 out of 118) postmenopausal women with Colles' fracture are identified as patients with established osteoporosis using DXA T-score -2 SD for BMD measured at either spine or hip.

**Table 1:** Anthropometric variables and DXA T-score. Homogeneity in anthropometric and BMD and DXA T-score in patients with established osteoporosis compared between Alendronate group and placebo control group (Data: mean ± SD)

Parameters	Alendronate group	Control group	p value
Number of subjects	22	25	/
Age (years)	60.7 ± 6.4	59.1 ± 6.3	0.387
Years since menopause (YSM) (years)	11.2 ± 7.5	9.1 ± 5.7	0.290
Height (m)	154.3 ± 5.9	153.9 ± 5.3	0.825
Weight (kg)	54.3 ± 9.5	56.8 ± 8.5	0.358
Body mass index (BMI)	22.7 ± 3.0	23.9 ± 2.5	0.173
Spine T-score	-2.21 ± 0.78	-2.20 ± 0.81	0.970
Hip T-score	-1.53 ± 1.03	-1.70 ± 0.51	0.508

### BMD at baseline and its changes compared between two study groups

**Figure 1:**
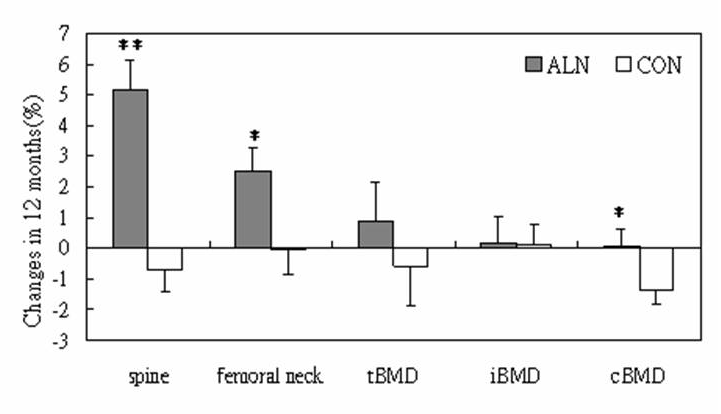
Percentage changes of bone mineral density after 12-months treatment compared between Alendronate (ALN) and placebo control group (CON) (Data are in mean ± SE). *: p < 0.05; **: p < 0.01.

Table 2 summarizes the baseline BMD and its changes at 6 and 12 months measured at spine and femoral neck by DXA and at non-fracture distal radius by pQCT. The percentage difference is also shown in Figure 1. No difference is shown for the baseline BMD between Alendronate group and control group. There is a significant increase in BMD in Alendronate group, with on average 5.1% at spine and 2.5% at femoral neck after 12 months treatment. Slight reduction of BMD is found at both spine (0.7%) and femoral neck (0.1%) in placebo control group after 12 months intervention, however without statistical significance (p > 0.05, for both). The percentage increase of BMD at distal radius of the Alendronate group is milder as compared with that found at spine and femoral neck after 12 months treatment, with 0.9%, 0.2% and 0.1% in tBMD, iBMD and cBMD, respectively. Only the increase in tBMD is found statistically significant as compared with its baseline (p < 0.05). Control group shows no change or slightly decreased BMD at distal radius (p > 0.05). DXA T-score is improved in Alendronate group, on average from -2.2 at baseline to -1.9 after 12-month intervention (p < 0.01).

**Table 2:** BMD data at baseline, 6- and 12-months. Comparison of BMD at baseline and its changes at 6- and 12-months in patients with established osteoporosis compared between Alendronate group and placebo control group (Data: mean ± SD)

BMD measurements		Placebo control group (n = 25)				Alendronate group (n = 22)			
		Baseline	6-month	12-month	% difference ^+^	Baseline	6-month	12-month	% difference ^+^

DXA (g/cm2)	Spine (L2-L4)	0.718 ± 0.101	0.709 ± 0.105	0.710 ± 0.102	-0.7 ± 3.3	0.719 ± 0.097	0.743 ± 0.101 *	0.756 ± 0.094 **	5.1 ± 4.2 ^a^
	Femoral Neck	0.631 ± 0.060	0.633 ± 0.053	0.632 ± 0.055	-0.1 ± 4.1	0.653 ± 0.121	0.658 ± 0.129	0.670 ± 0.129 **	2.5 ± 3.2 ^b^
pQCT (mg/cm3) Distal radius	tBMD	138.6 ± 29.3	135.3 ± 31.5	137.3 ± 31.9	-0.6 ± 6.4	126.8 ± 46.4	130.4 ± 44.3 *	130.8 ± 44.5 *	0.9 ± 5.1
	iBMD	424.5 ± 73.0	421.0 ± 75.6	429.9 ± 82.3	0.1 ± 3.2	388.1 ± 79.4	383.4 ± 79.6	385.4 ± 91.0	0.2 ± 3.8
	cBMD	1124.2 ± 172.9	1070.7 ± 289.3	1113.0 ± 178.4 **	-1.4 ± 2.2	1084.3 ± 202.8	1024.7 ± 329.8	1077.6 ± 203.3	0.1 ± 2.4 ^b^

+: percentage compared between baseline and 12-month

* p < 0.05; **p < 0.01: compared with baseline

a: p < 0.01; b: p < 0.05: compared for changes in BMD between Alendronate and placebo control group

### Bone turnover biochemical markers

**Figure 2:**
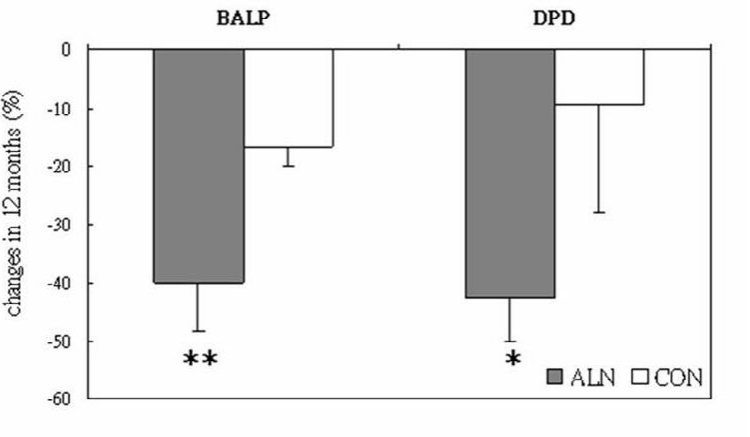
Percentage changes of Bone Alkaline Phosphate (BALP) and Deoxypyridoline (DPD) level after 12-month treatment compared between Alendronate (ALN) and placebo control group (CON) (Data are in mean ± SE). *: p < 0.05; **: p < 0.01.

Table 3 summarizes bone forming serum alkaline phosphatase (BALP) and urinary Deoxypyridoline (DPD) in both Alendronate group and control group at baseline and their changes at 6-months and 12-months. The percentage difference is also shown in Figure 2. BALP and DPD level decreases 39.9% and 42.6% respectively (p < 0.01 for both) in Alendronate group after 12 months treatment while the control group shows 16.7% decrease in BALP level (p < 0.01) and no change in DPD level. The changes in both BALP and DPD are found significantly different between Alendronate treatment and placebo control group (p < 0.05 and p < 0.01, respectively).

**Table 3:** Bone formation and resorption markers. Bone Alkaline Phosphate (BALP) and Deoxypyridinoline (DPD) level in patients with established osteoporosis compared between Alendronate group (ALN) (n = 22) and placebo control group (CON) (n = 25) at baseline and follow-up at 6- and 12 months (Data: mean ± SD)

Biochemical Markers	Group	Baseline	6-month	12-month	% difference^+^
BALP	ALN	62.27 ± 16.86	40.33 ± 9.98 **	34.35 ± 9.87 **	-39.9 ± 33.6^a^
	CON	66.17 ± 14.91	56.72 ± 18.24 **	55.13 ± 15.76 **	-16.7 ± 14.5
DPD	ALN	50.82 ± 35.10	25.11 ± 18.65 **	23.03 ± 14.72 **	-42.6 ± 30.4^b^
	CON	33.70 ± 20.28	40.11 ± 36.92	27.28 ± 10.95	-9.4 ± 26.7

+: percentage difference compared between baseline and 12-month

a: p < 0.01; b: p < 0.05: significant difference in BALP found between ALN and CON group (independent t-test)

*p < 0.05; ** p < 0.01: compared with baseline (paired t-test)

### Dropout and fracture cases

There are 13.6% (3 out of 22 subjects) and 4% (1 out of 25 subjects) patients dropped out in Alendronate and control group during 12 months intervention, respectively. The main reasons of dropout are due to loss contact in follow up measurement. None of them dropped out due to uncomfortable feeling of stomach development after Alendronate treatment. Only one ankle fracture is recorded in control group during 12-month intervention as a result of fall.

## Discussion

This study was designed to evaluate 12-month Alendronate treatment effects on BMD in postmenopausal women with established osteoporosis, i.e. patients with both low-energy Colles' fracture and BMD values below -2SD of DXA T-score measured either at spine or at hip and with. The use of -2SD of DXA T-score as intervention thresholds for drug treatment was chosen based on treatment efficacy and cost-effectiveness recommended in the past [5,11,14,15].

The major findings of the present study were that firstly 40% of postmenopausal women were found with DXA T-score lower that -2SD at either lumbar spine or femoral neck; and secondly there was 0.1%–5.1% overall increase in BMD at both axial and appendicular skeletons after 12-montts Alendronate treatment. The Alendronate treatment effect was revealed greater in weight bearing bones (5.1% at lumbar spine and 2.5% at femoral neck) that non-weight bearing non-dominant distal radius (0.9% in tBMD, 0.2% in iBMD, and 0.1% in cBMD). This finding was consistent with that of other studies using either Alendronate or estrogen treatments in both Caucasian [16,17] and Chinese population [8,9]. When comparison was made for non-weigh bearing distal radius, there was only very mild increase in trabecular BMD at distal radius in Alendronate group as compared with placebo-control group. This might suggest that Alendronate was not effective to increase BMD at the non-weight bearing skeletons.

Alendronate is a known strong inhibitor of bone resorption mainly by inducing apoptosis and impairing the function of osteoclasts as well as by preventing the apoptosis of osteocyte and osteoblast [18,19]. Alendronate was reported to suppress the high bone turnover and increase BMD in osteoporosis patients in various ethic populations [8,20,21]. In the present study, we also showed the same underling mechanism of Alendronate in prevention of bone loss and/or increase of BMD in postmenopausal Chinese women with established osteoporosis. BALP and DPD were used as a couple of bone turnover biochemical markers and its turnover was suppressed significantly in Alendronate group, slightly more in bone-resorbing marker DPD as compared bone forming marker BALP at the first 6 months and continued over the 12 months intervention. This result was also similar to previous reports for the same reference population [8,9] or other ethnic groups [13,20,21].

Interestingly, the present study also showed that calcium supplement alone in placebo control was also able to retard bone loss at both axial and appendicualr skeletons in postmenopausal women with established osteoporosis as compared with significant bone loss in postmenopausal women of the same reference populations without any treatments reported previously [12,22]. On the other hand, the calcium placebo effects in the present study were found comparably higher than the reported one in Caucasians [13,20,21]. This might be associated with the fact that the average calcium intake of Chinese women in Hong Kong (less than 800 mg/day) was notably lower than that of Caucasian women (around 1300 mg/day) [8,9,20].

To identify patients with established osteoporosis for prevention is clinically important as the occurrence of osteoporotic fractures increase with advancing age, in general with a sequence of Colles' fracture, vertebral fracture, and hip fractures. Logistically, to identify osteoporotic patients with low-energy Colles' fracture should be therefore more relevant for early interventions for prevention of subsequent osteoporotic fractures as spine and hip [1,2,4,14,23,24]. The current study only recorded one ankle fracture in the control group. Such intervention study with duration of 12-months was rather short and therefore, we did not use prevention as the end-point in evaluation of treatment effects. At present, it was still uncertain how long the patient should remain on drug therapy for prevention or treatment of osteoporosis and prevention of osteoporotic fractures. However, short- or mid-term drug intervention for 1–2 years might be effective for not only improving BMD but also preventing current and late fractures as there was no any acceleration in bone loss after discontinuing the drug treatment found by others [21,23,25,26].

In the present study, the treatment tolerability for the anti-osteoporotic drugs was reflected partially by the dropout rate. We recorded 13.6% and 4% dropouts in Alendronate and control group during 12 months intervention, respectively. However, none of them dropped out due to discomfort of stomach after Alendronate treatment. This data was similar to the Alendronate studies on prevention of osteoporosis in the same reference population [8,9]. In summery, this 12-months intervention study demonstrated the treatment effect of Alendronate on osteoporosis at both axial and appendicualr skeletons in Chinese postmenopausal women with low-energy Colles' fracture, with more effect on the weight-bearing spine and hip than the non-weigh bearing distal radius. Such effects were explained by significant suppression in bone turnover evaluated biochemically.

## Competing interests

The institution of the authors has received funding from the Hong Kong Health Services Research Committee. Authors do not have any potential conflicts of interests related to this work
